# Identification of tumor associated immune responses against brachyury, a transcription factor and driver of EMT, in chordoma patients receiving a yeast-brachyury vaccine (gi-6301)

**DOI:** 10.1186/2051-1426-2-S3-P148

**Published:** 2014-11-06

**Authors:** Renee N Donahue, Italia Grenga, Lauren Lepone, James L Gulley, Christopher R Heery, Ravi A Madan, Timothy C Rodell, Jeffrey Schlom, Benedetto Farsaci

**Affiliations:** 1 Laboratory of Tumor Immunology and Biology, CCR, NCI, NIH, Bethesda, MD, USA; 2 CCR, NCI, NIH, Bethesda, MD, USA; 3 Genitourinary Malignancies Branch, CCR, NCI, NIH, Bethesda, MD, USA; 4 GlobeImmune, Inc, Louisville, KY, USA

## Purpose

Brachyury is a tumor-associated antigen and transcription factor that drives the epithelial-to-mesenchymal transition (EMT) in human carcinomas. The aim of this study was to assess whether patients with chordoma, a rare tumor of the notochord that over-expresses brachyury, can elicit a brachyury-specific T cell response following yeast-Brachyury vaccination.

## Methods

An expansion cohort of 7 patients with chordoma, enrolled in the Phase I clinical trial "Open Label Study to Evaluate the Safety and Tolerability of GI-6301 (Whole Heat-Killed Recombinant yeast Modified to Express Brachyury Protein) in Adults with Solid Tumors", NCT01519817, were assessed for brachyury-specific T cell responses. Patients received 40 yeast units of vaccine every 2 weeks, and monthly dosing following restaging at day 85. PBMCs from pre- and post-vaccination were cultured in a 7-day in vitro stimulation (IVS) with overlapping 15-mer peptides of the brachyury protein that was encoded in the vaccine, and IL7/IL15. Following the IVS, cells were rested for 4 days, and then re-stimulated with 15-mer peptides. HLA 15-mers and a mixture of 9-mer to 15-mers of CMV, EBV, Flu, and Tetanus Toxin (CEFT) served as negative and positive controls, respectively. Brachyury-specific T cell responses were analyzed by flow-cytometry intracellular staining (ICS) of CD4 and CD8 T lymphocytes for the cytokines IFN-γ, TNF, and IL-2, and the perforin/granzyme marker CD107a. Cells positive for ≥2 cytokines were considered multipotent T lymphocytes, while cells co-expressing at least one cytokine and positive for CD107a were classified as having a cytokine/lysis association. A patient was considered an immune responder if, after brachyury 15-mer IVS, the frequency of T lymphocytes positive for a cytokine or CD107a post-vaccine was >50% compared to both (1) pre-vaccine values and (2) HLA control post-vaccine.

## Results

43% of patients (3/7) had a response to brachyury, with 2 having only a CD4 response, and 1 having both a CD4 and CD8 response. Of the 3 immune responders, 1 had only a single cytokine response, 1 had multipotent T lymphocytes, and 1 had a cytokine/lysis association.

## Conclusions

These findings show for the first time that chordoma patients immunized with brachyury, a tumor associated antigen and transcription factors that drives EMT, can develop a brachyury-specific T cell immune response. These results warrant further studies using this vaccine in additional chordoma patients.

